# The impact of intraoperative goal-directed fluid therapy on complications after pancreaticoduodenectomy

**DOI:** 10.1016/j.amsu.2018.10.018

**Published:** 2018-10-16

**Authors:** Jesse K. Sulzer, Amit V. Sastry, Lauren M. Meyer, Allyson Cochran, William C. Buhrman, Erin H. Baker, John B. Martinie, David A. Iannitti, Dionisios Vrochides

**Affiliations:** Division of HPB Surgery, Department of Surgery, Carolinas Medical Center, Charlotte, NC, USA

**Keywords:** Fluid therapy, Gastric emptying, Pancreatectomy, Pancreaticoduodenectomy, Pancreatic leak, Postoperative complications, Stroke volume

## Abstract

**Introduction:**

Optimal fluid balance is critical to minimize anastomotic edema in patients undergoing pancreaticoduodenectomy. We examined the effects of decreased fluid administration on rates of postoperative pancreatic leak and delayed gastric emptying.

**Methods:**

Retrospective study of 105 patients undergoing pancreaticoduodenectomy at a single institution from January 2015 through July 2016. Stroke volume variation (SVV) was tracked and titrated during the procedure. A comparative analysis of postoperative complications was performed between patients with a median SVV < 12 during the extirpative and reconstructive phases of the procedure compared with patients with an SVV ≥ 12.

**Results:**

Of 64 patients who met selection criteria, 42 (65.6%) had a SVV < 12 and 22 (34.4%) had a SVV ≥ 12. Patients with an SVV ≥ 12 during the extirpative phase of the procedure had lower rates of postoperative pancreatic leaks compared to patients with an SVV < 12 (5.9% vs 21.3%)). Patients with an SVV ≥ 12 during the extirpative phase had lower rates of postoperative delayed gastric emptying compared to patients with an SVV < 12 (41.2% vs 46.8%).

**Conclusion:**

Goal-directed fluid restriction before the reconstructive phase of pancreaticoduodenectomy may contribute to lower postoperative rates of pancreatic leak and delayed gastric emptying.

## Highlights

• Optimal fluid balance is critical to minimize anastomotic edema in patients undergoing pancreaticoduodenectomy.
• Fluid restriction before the reconstructive phase of pancreaticoduodenectomy may decrease pancreatic leak and delayed gastric emptying.
• Non-invasive hemodynamic monitoring systems offer the anesthesiologist a method to titrate intravascular fluid balance.
• Goal-directed fluid management is a recent clinical practice during pancreatic surgery that may help decrease postoperative complications.

## 1 Introduction

Short-term morbidity after pancreatectomy remains a challenging issue. Postoperative complications such as pancreatic leak and delayed gastric emptying occur in nearly half of patients; some may be potentially life threatening [1,2]. Patients who have these complications often have a protracted hospital course, a longer interval to adjuvant systemic therapy, poorer quality of life, poorer nutrition, and are at an increased risk for catastrophic intra-abdominal hemorrhage [3].

Pancreaticoduodenectomy, perhaps the most recognized pancreatectomy, has been fraught with these complications throughout the history of the operation [4]. The procedure has since undergone several technical modifications to mitigate the considerable complication rate [5,6].

Improvements in the pancreatico-jejunal anastomotic technique and utilization of a round ligament flap or omental wrappings have shown some benefit in reducing pancreatic leak [[7], [8], [9]]. Modifications of the enteric anastomosis have yielded some modest improvements in the rate of delayed gastric emptying [10]. Patient outcomes have been favorably impacted by initiatives to perform these complex procedures at high-volume centers, as well as advances in minimally-invasive techniques, which have gradually been incorporated into surgical practice [11,12].

Despite these interventions, morbidity after surgery remains considerable with overall estimates ranging from 20% to 30% of patients [13]. Postoperative pancreatic leaks affect 11%–18% of patients, whereas delayed gastric emptying affects about 20% of patients [[14], [15], [16]].

Recently, Enhanced Recovery After Surgery (ERAS^®^) pathways have gained popularity in abdominal surgery [17]. Many of these pathways endorse restricted perioperative intravenous fluid administration, which may play a role in reducing anastomotic edema and postoperative fluid compartment shifts [18,19]. Additionally, there has been a trend toward goal-directed fluid resuscitation [20,21]. The development of non-invasive hemodynamic monitoring systems offers the anesthesiologist a method to titrate intravascular fluid balance [22,23]. Specifically, it allows continuous monitoring of stroke volume variation (SVV) during the operation by using an arterial line [24]. Stroke volume variation reflects changes in left ventricular output secondary to intrathoracic pressure changes induced during mechanical ventilation. In relation to the Frank-Starling curve, which equates stroke volume to cardiac preload, SVV serves as a marker of position along the curve [25]. By monitoring the change in stroke volume noted during different phases of the respiratory cycle in mechanically ventilated patients, the anesthesiologist can assess fluid status and accurately predict an individual patient's responsiveness to fluid administration [26,27].

Goal-directed fluid management is a relatively new clinical practice during pancreatic surgery that may be beneficial to patients if it contributes to fewer postoperative complications. In this study, the authors propose that reducing the volume of intravascular fluid administered during pancreaticoduodenectomy may result in a decrease in the number of patients with postoperative pancreatic leak and/or delayed gastric emptying. We hypothesized that patients undergoing pancreaticoduodenectomy with a SVV titrated to greater than or equal to 12 may have fewer postoperative complications compared with patients with a SVV titrated to less than 12.

## 2 Methods

Following approval by the local Institutional Review Board, a single institution review of 105 patients who consecutively underwent pancreaticoduodenectomy at Carolinas Medical Center from January 2015 through June 2016 was conducted retrospectively. Study data were collected and managed using the Research Electronic Data Capture (REDCap) [28]. All patients were seen preoperatively in the department clinic setting, and indications for pancreaticoduodenectomy were for pancreatic adenocarcinoma, neuroendocrine tumors, chronic pancreatitis, non-adeno malignancy, and other benign. Patients were excluded if they had any of the following during surgery: venous resection and reconstructive involving the portal venous system; estimated blood loss exceeding 2 L; high dose steroid administration; use of irreversible electroporation for margin enhancement; lack of SVV equipment or inconsistent SVV recordings; use of the robotic surgical system.

Operations were performed at a high-volume center by one of four fellowship-trained hepatopancreatobiliary surgeons. All patients underwent an open approach for pancreaticoduodenectomy and received the standard ERAS pathway [29]. The pancreaticojejunostomy was performed in duct to mucosa fashion. Pylorus preservation was performed based on intraoperative factors and surgeon preference. Non-invasive hemodynamic monitoring was performed by the anesthesiologist by using the Edwards Vigileo™ system with an existing arterial line. Stroke volume variation was recorded every 15 minutes during the operation. Anesthesiologists followed a policy of fluid restriction, with an SVV goal of 12. Fluid administration was adjusted based on intraoperative changes made by the anesthesiologist per a standardized protocol (Fig. 2).

**Fig. 1:**
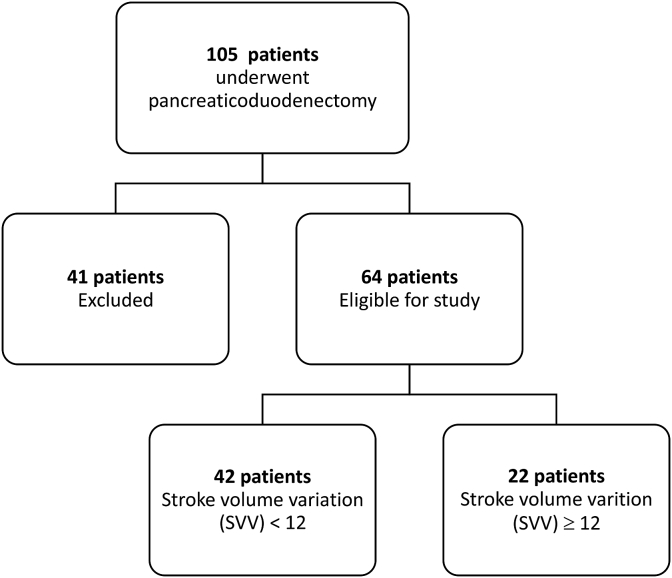
Flow diagram of patient selection.

**Fig. 2:**
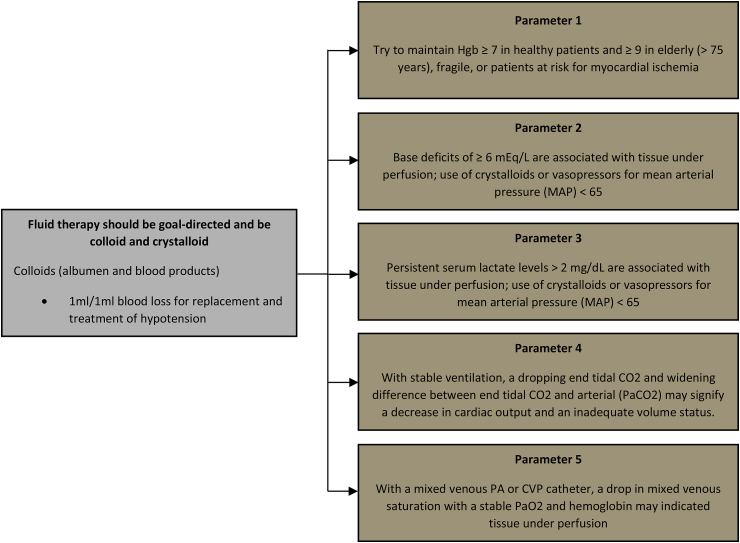
Recommended anesthesia flowchart for fluid management during pancreaticoduodenectomy.

The following demographic data were obtained for each patient: age, patient weight, American Society of Anesthesiology (ASA) score, total intravenous fluids administered intraoperatively, number of units of packed red blood cells transfused, total fluids adjusted for weight, total intravenous fluids administered divided by the sum of the total calculable fluid losses, perioperative epidural catheter placement, and extubation in the operating room. Stroke volume variation recorded during the procedure was defined as follows:

• SVV - median stroke volume variation throughout the procedure.
• SVV_ext_ - median stroke volume variation preceding the last 2 h of the procedure. This represents the “extirpative phase” of the operation.
• SVV_rec_ - median stroke volume variation during the last 2 h of the procedure. This represents the “reconstructive phase” of the operation.

Primary outcomes recorded for each patient were pancreatic leak and delayed gastric emptying. Pancreatic leak was defined according to the international study group for pancreatic fistulas: “an external fistula with a drain output of any measurable volume after postoperative day three with an amylase level greater than three times the upper limit of the normal serum value.” [30] Delayed gastric emptying was defined clinically as persistent postoperative emesis requiring nasogastric tube placement, prokinetic agents, or hospital readmission for endoscopic gastrostomy placement.

After obtaining these variables, the patients were evaluated based on the median SVV, SVV_ext_, and SVV_rec_. We compared patients with SVV, SVV_ext_ and SVV_rec_ < 12 to those with SVV, SVV_ext_ and SVV_rec_ ≥12 for differences in postoperative outcomes: pancreatic leak and delayed gastric emptying. We chose an SVV value of greater than 12 to represent a “dry” state because previous studies have shown that this value represents decreased fluid administration [31,32].

### 2.1 Statistics

Patient and operative characteristics were compared between the SVV < 12 and SVV ≥ 12 groups. Variables were tested for normality. Continuous variables were reported as median values and analyzed using the Mann-Whitney test statistic. Nominal and dichotomous variables were reported as percentages and analyzed using the Chi-square test statistic. Statistical significance was considered at *P* < .05. Statistical analysis was performed using STATA (STATACorp, v.13).

## 3 Results

Sixty-four patients meet selection criteria and were included in the analysis. Of these 64 patients, 42 had an SVV <12, and 22 had an SVV ≥12 (Fig. 1). Forty-seven of these 64 patients had a SVV_ext_ and SVV_rec_ < 12, whereas the remaining 17 patients had a SVV_ext_ and SVV_rec_ ≥ 12.

There was no statistically significant differences between patients who had a SVV <12 compared with patients who had a SVV ≥12 in terms of: median age, ASA score, etiological classification, pancreatic texture, patient weight, total fluids administered, packed red blood cells administered, total intravenous fluids administered adjusted for weight, total fluids administered divided by the sum of the estimated blood loss, urine output, and any other calculable fluid losses, perioperative epidural catheter placement, or extubation in the operating room (Table 1).

**Table 1:** Median stroke volume variation and patient demographics.

	Median SVV <12 (n = 42)	Median SVV ≥ 12 (n = 22)	*P* value
**Age, years**^a^	63 (32–84)	63.5 (24–82)	.651
**Etiological classification**			.737
Pancreatic adenocarcinoma	31 (73.8)	18 (81.8)	
Neuroendocrine tumor	2 (4.8)	2 (9.1)	
Chronic pancreatitis	4 (9.5)	1 (4.6)	
Non-adenocarcinoma malignancy	4 (9.5)	1 (4.6)	
Other benign	1 (2.4)	0	
**ASA score (n)**			
2	4 (9.5)	2 (9.1)	.955
3	35 (83.3)	17 (77.3)	.555
4	3 (7.1)	3 (13.6)	.397
**Pancreatic texture**^b^^,^^c^			.615
**Soft**	24 (57.1)	14 (63.6)	
**Firm/hard**	18 (42.9)	8 (36.4)	
**Weight, kg**^a^	68.5 (53.9–104)	76.5 (45.8–88.4)	.955
**Total fluids, mL**^a^	2650 (1500–5400)	2450 (1200–6301)	.783
**PRBC, mL**^a^	0	0	.305
**Total fluids weight-adjusted, mL/kg**^a^	39.3 (18.9–85.7)	39.7 (13.6–109.2)	.832
**Case I/O**^a^	2.5 (1.4–12)	2.5 (1.5–11.7)	.816
**Estimated blood loss, mL**^a^	400 (75–1800)	475 (100–1700)	.353
**Urine output, mL**^a^	507.5	467.5	.326
**Epidural catheter placed**^b^			
Yes	33 (78.6)	12 (86.4)	.448
**Extubation**^b^			
Yes	38 (90.5)	17 (77.3)	.149

Abbreviations: SVV: stroke volume variation; ASA Score: American Society of Anesthesiology score; kg: kilograms; total fluids: total intravenous fluids administered during the operation; mL: milliliters; PRBC: packed red blood cells administered, case I/O: total fluids administered divided by the sum of the estimated blood loss, urine output, and any other calculable fluid losses; EBL: estimated blood loss, epidural placement: perioperative epidural catheter placement; extubation: removal of endotracheal tube in the operating room.

a Value expressed as median (range).

b Value expressed as number (percentage).

c Subjective assessment of pancreatic texture by the surgeon.

**Fig. 3:**
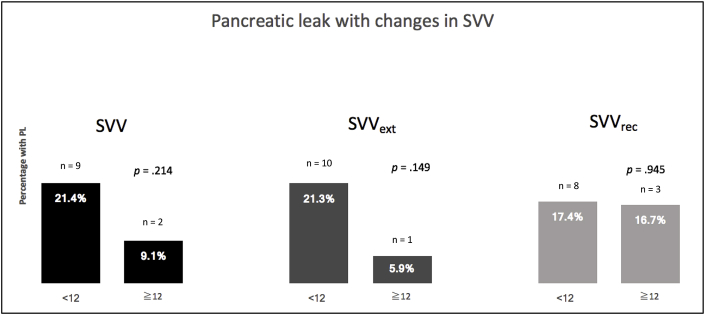
Rates of pancreatic leak between the two groups based on stroke volume variation (SVV) titration.

The rates of pancreatic leak were lower for patients who had a median SVV ≥12 compared with patients who had a median SVV <12. Although this numerical decrease in the rates of pancreatic leaks was observed for all three groups: SVV, SVV_ext_, and SVV _rec_, this finding was not statistically significant: SVV <12 vs. SVV ≥12: n = 9 and n = 2; SVV_ext_ < 12 vs. SVV ≥12: n = 10 and n = 1; SVV_rec_ < 12 vs. SVV ≥12: n = 8 and n = 3 (*P* = .214, .149, and 0.945, respectively) (Fig. 3). Pancreatic texture, as assessed by the surgeon intraoperatively, was similar between groups (Table 1).

**Fig. 4:**
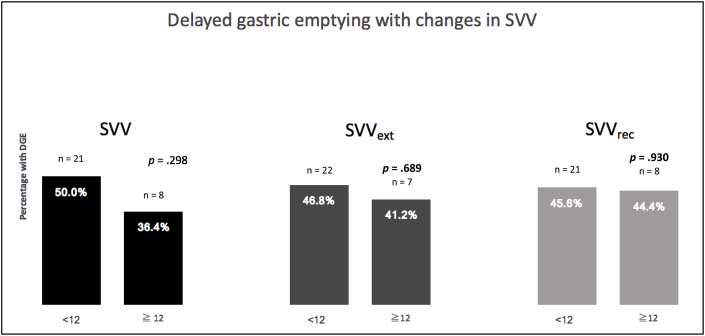
Rates of delayed gastric emptying between the two groups based on stroke volume variation (SVV) titration.

The rates of delayed gastric emptying were lower for patients who had a median SVV ≥12 compared with patients who had a median SVV <12. Although this numerical decrease in the rates of delayed gastric emptying was observed for all three groups: SVV, SVV_ext_, and SVV _rec_, this finding was not statistically significant: SVV <12 vs. SVV ≥12: n = 21 and n = 8; SVV_ext_ < 12 vs. SVV ≥12: n = 22 and n = 7; SVV_rec_ < 12 vs. SVV ≥12: n = 21 and n = 8, (*P* = .298, .689, and 0.930, respectively) (Fig. 4).

## 4 Discussion

We aimed to determine whether goal-directed fluid administration targeted to a SVV ≥12 during pancreaticoduodenectomy was associated with fewer postoperative complications: pancreatic leak and delayed gastric emptying. To understand the role of SVV and postoperative complications, we excluded patients with known confounding variables for anastomotic complications, such as an estimated blood loss exceeding 2 L and perioperative steroid use. The study evaluated a relatively homogenous subset of patients during an 18 month period. Our findings showed that patients who had an SVV ≥12 had fewer postoperative complications compared with patients who had an SVV <12. This was particularly evident for patients who had a SVV_ext_ ≥ 12, as the rate of pancreatic leak was 5.9% compared to a rate of 21.3% for patients who had a SVV_ext_ < 12, although this difference was not statistically significant.

To our knowledge, this was the first study to evaluate short-term outcomes after pancreaticoduodenectomy regarding SVV titration. As previously stated, the authors believe that the SVV_ext_ calculation best captures the volume administrated in the extirpative phase of the procedure. Thus, it could be postulated that increased fluid administration in this phase may result in increased intestinal mucosal edema at the time of the reconstructive phase [19]. Additionally, there was a minimal difference in the rates of postoperative pancreatic leak and delayed gastric emptying in patients with SVV_rec_ < 12 compared with SVV_rec_ ≥ 12. This finding may be related to an excess fluid administration in the extirpative phase with mucosal edema already present during the reconstruction.

Precise fluid resuscitation has been studied extensively in other fields and has been shown to be particularly applicable to colorectal surgery [26,27]. Data shows that excessive fluid administration can lead to bowel edema at the site of anastomosis resulting in increased rates of anastomotic breakdown [33,34]. Until recently, this information has not been evaluated in patients undergoing pancreaticoduodenectomy. Weinberg and colleagues retrospectively evaluated 150 consecutive patients undergoing pancreaticoduodenectomy and found that those with complications had a higher median volume of intravenous therapy, poorer cumulative positive fluid balance, and longer median length of stay [35]. Additionally, Kulemann and colleagues retrospectively evaluated 553 patients undergoing pancreaticoduodenectomy and reported higher postoperative complication rates in the group of patients who received greater amounts of fluid during the intraoperative and postoperative periods [33]. These studies provide additional validity to the hypothesis presented here; however, some important differences exist. The volumes of intraoperative fluid administered in prior studies was much higher than the median volumes reported here. Kulemann et al. set a cut-off point of greater than 6 L of intraoperative fluid [33]. Both groups in our study had a median intraoperative fluid volume of less than 2700 mL.

An additional important difference between this and prior studies was the focus on SVV rather than volume alone. There were no differences in total volume administered between groups in this study. A meta-analysis by Huang et al. evaluating the impact of perioperative fluid administration on early outcomes following pancreaticoduodenectomy failed to demonstrate a difference in outcomes based on fluid volume alone leading the authors to suggest a strategy of tailoring fluid administration to the patient's physiology rather than an absolute fluid goal [36]. Stroke volume variation allows for continuous monitoring and adjustment of fluid balance throughout the procedure on an individual basis and may affect other factors not directly captured by evaluating overall fluid volume. A prospective, randomized study using SVV in high-risk patients undergoing abdominal surgery suggested that improved hemodynamic stability during the procedure was likely a factor that contributed to improved patient outcomes [37].

Intraoperative fluid optimization with SVV could be associated with fewer brief hypotensive episodes. Although this may not be evidenced by total fluid volumes, it may contribute as another source of improved outcomes. The potential importance of adjusting to each patient's physiology rather than overall fluid goals could be supported by our findings that a difference in complication rates were observed between groups without a significant difference in fluid volumes. Timing of fluid administration may prove another key factor as hypothesized in this study. Limiting fluid administration prior the reconstructive phase as guided by SVV may result in a similar total volume administered but decreased tissue edema while performing the anastomoses.

Although many efforts were made to preserve homogeneity between the groups studied, we excluded patients who underwent a robotic-assisted pancreaticoduodenectomy. The literature reports similar outcomes between a robotic and open approach for pancreaticoduodenectomy, but it remains unclear if this introduced bias. Additionally, the effect of pneumoperitoneum on SVV monitoring is an important consideration. Wajima and colleagues demonstrated statistically significant changes in SVV within 5 min upon initiation and withdrawal of pneumoperitoneum [34]. In this study, there was no difference in outcomes between the two groups when robotic pancreaticoduodenectomy cases were removed from the analysis.

This study had a few key limitations. Several patients were excluded due to the high volume of complex pancreaticoduodenectomy procedures performed at our institution; many of which included portal venous resection and irreversible electroporation for vascular margin enhancement. These cases are often associated with high blood loss (up to 1500 mL) and aggressive fluid resuscitation could ensue at any point in the operation based on preference of the surgeon and/or anesthesiologist [38]. Statistical significance was not achieved with this data set likely due to type II error. A post-hoc analysis revealed that the number of patients that would have been needed to evaluate statistical significance in this study was 146.

Our results support the principles of perioperative fluid restriction and address a means to establish the appropriate amount of fluid administration for select patients undergoing pancreaticoduodenectomy; they also suggest an important role for the timing of fluid administration during pancreaticoduodenectomy. Although our findings do not support achange in perioperative care because there was not a statistically significant difference in rates of postoperative complications, further study of goal-directed fluid restriction may yield better outcomes. Reducing primary complications from pancreaticoduodenectomy could translate to fewer postoperative interventions such as percutaneous abscess drainage, percutaneous endoscopic gastrostomy placement, or an emergent reoperation or arterial embolization for gastroduodenal hemorrhage [39,40]. Additionally, the goal for many patients who undergo pancreaticoduodenectomy for pancreatic adenocarcinoma is to begin adjuvant chemotherapy [35]. Decreasing complications after pancreaticoduodenectomy may offer patients a shorter length of stay and prompt initiation of adjuvant therapy.

Future research on goal-directed fluid resuscitation in pancreaticoduodenectomy should be directed towards a proof-of concept study, which evaluates the optimal fluid therapy based on the phase of the operation: 1) titrate SVV to maintain a “dry” state (*e.g.*, SVV > 12) before the anastomosis, and 2) titrate SVV for euvolemia after the anastomosis. A well-powered study constructed in prospective fashion could allow for a better answer to the clinical question of whether a non-uniform approach to intraoperative fluid therapy helps reduce postoperative complications. This study followed the preferred reporting of case series in surgery according to the PROCESS guidelines [41].

In summary, our findings introduce a systemic concept to support the principle of perioperative fluid restriction for select patients undergoing pancreaticoduodenectomy. The use of ERAS pathways in pancreatic surgery may further allow for evaluation of clinical outcomes, in the setting of intraoperative and postoperative fluid restriction.

## 5 Conclusion

Titration of intraoperative SVV ≥12 may lead to decreased rates of pancreatic leaks and delayed gastric emptying after pancreaticoduodenectomy. This outcome may suggest that goal-directed fluid restriction results in less anastomotic edema.

## Provenance and peer review

Not commissioned, externally peer reviewed.

## Ethical approval

Research ethics approval was provided by the Institutional Review Board of Carolinas HealthCare System (IRB File #: 06-12-34E).

## Sources of funding

No funding sources.

## Author contribution

Study design: AS, LC, WB, DV.

Data collection: AS, EB, JM, DI.

Interpretation: JS, AS, DV, JM, DI.

Statistical analysis: AC, JS.

Writing first draft of manuscript: JS, AS, JS, DV.

Critical review and approval of manuscript: All authors.

## Conflicts of interest

No conflicts to declare.

## Research registration number

NCT03699917.

## Guarantor

Dionisios Vrochides, MD, PhD, Division of Hepatopancreatobiliary Surgery, Department of Surgery, Carolinas Medical Center, 1000 Blythe Boulevard, Charlotte, NC 28203, Email: dionisios.vrochides@atriumhealth.org.

## Appendix A Supplementary data

Process Checklist - Ann Med Surg revision Process Checklist - Ann Med Surg revision
